# Therapeutic potential of trazodone in trigeminal neuralgia based on inflammation and oxidative stress: an *in vitro* experimental study

**DOI:** 10.22514/jofph.2024.020

**Published:** 2024-06-12

**Authors:** Jun Yang, Junling Huang, Zhimin Pan, Xiao Wang

**Affiliations:** Department of Geriatrics, The First Affiliated Hospital of Zhejiang Chinese Medical University (Zhejiang Provincial Hospital of Chinese Medicine), 310003 Hangzhou, Zhejiang, China; Department of Geriatrics, Tongji Hospital, School of Medicine Tongji University, 200065 Shanghai, China

**Keywords:** Trigeminal neuralgia (TN), Trazodone, Inflammation, Oxidative stress, MAPK pathway

## Abstract

Trigeminal neuralgia (TN) is a debilitating condition affecting the patients’ life quality. New therapeutic approaches and novel drugs are required to treat TN. Trazodone being a serotonin antagonist and reuptake inhibitor (SARI) provides neuroprotection, however its role and underlying mechanism in TN *in vitro* or *in vivo *are not clear. This study was aimed to investigate the trazodone impact on glial BV-2 cells regarding TN. It was found that trazodone inhibited the BV-2 cells growth and suppressed the inflammation and oxidative stress in Lipopolysaccharide (LPS)-treated BV-2 cells. Trazodone treatment specifically decreased the levels of Tumor Necrosis Factor-alpha (TNF-α), Interleukin-6 (IL-6), Interleukin-1 beta (IL-1β) (*p *< 0.05), and Reactive Oxygen Species (ROS) (*p *< 0.01). Moreover, trazodone suppressed the Mitogen-Activated Protein Kinase (MAPK) pathway in LPS-treated BV-2 cells. These outcomes demonstrate that trazodone suppressed glial cell hyperproliferation, inflammation, and oxidative stress through MAPK pathway activation.

## 1. Introduction

Trigeminal neuralgia (TN) is a distressing condition affecting patients’ life quality. TN is diagnosed by the local pain in one or more branches of trigeminal nerve [1]. The pathology and TN mechanisms are attributed to neuropathic pain (NP), trigeminal ganglion (TG), central trigeminal spinal nucleus, and neuroinflammation with hyperalgesia through neuronal receptors activation [2]. However, TN underlying mechanism is yet not clear.

The glial cells activation in central nervous system (CNS) releases inflammatory cytokines to increase the pain hypersensitivity and participate in the pathogenesis of neuropathic pain [3, 4]. The targeted suppression of glial cell activation is thus a new potential therapeutic target for TN.

Trazodone is a serotonin antagonist and reuptake inhibitor (SARI) available since early 1970s to treat depression with or without anxiety [5]. Pertaining to neuroprotection, trazodone and gabapentin restore innate behaviors in chronic constrictive injury rats. Behaviors are minimized or even disappeared during the persistent nociception which suggest that the combination may also affect different pain components [6, 7]. Trazodone therapy protects neuron-like cells from inflammatory damage by blocking Nuclear Factor kappa-light-chain-enhancer of activated B cells (NF-κB), p38 and Nuclear Factor kappa-light-chain-enhancer of activated B cells (JNK) [8]. Acute low-dose trazodone restores glutamate release efficiency and mGlu2/3 autoreceptor injury in the spinal cord of chronic sciatic ligation rats [9]. Trazodone increases 5-Hydroxytryptamine (5-HT) extracellular levels through the dual mechanism involving 5-HT transporter and 5-HT receptor [10, 11]. Trazodone manages conditions such as depression, anxiety, and insomnia in humans to reflect its Central Nervous System (CNS) related activity. Its role in treating TN has not been widely studied [9, 10, 11].

MAPK signaling pathway is essential for cellular processes including cell growth and stress response [6]. MAPK pathway activation in glial cells can regulate cell proliferation to maintain CNS homeostasis, however, its excess can cause neuroinflammation [6]. Furthermore, MAPK pathway mediates response to oxidative stress and influences the balance between reactive oxygen species (ROS) production and antioxidant defense [6].

This study is planned to explore the trazodone impact on glial cells. Trazodone inhibits glial cell hyperproliferation, inflammation, and oxidative stress by activating MAPK signaling.

## 2. Materials and methods

### 2.1 Cell culture and treatment

Mice glial cell line BV-2 was obtained from American Type Culture Collection (ATCC), and cultured in Dulbecco’s Modified Eagle Medium (DMEM) complete medium (11965092, Gbico, Grand Island, NY, USA) with 5% CO_2_ at 37 °C. LPS (100 ng/mL, #916734, Merck, Darmstadt, Germany) was treated with BV-2 cells for 24 h to construct TN cell model. Furthermore, trazodone (BP590, Sigma, St. Louis, MO, USA) was treated with BV-2 cells at 0, 0.5, 1 and 2 μM concentrations for 24 h. The TN model was constructed according to the precise study [2].

### 2.2 Cell viability assays

BV-2 cells were seeded into 96-well plates and kept at 37 °C. Cells after the mentioned treatment of 24 h were subsequently treated with cell counting kit-8 (CCK-8) reagent (C0038, Beyotime, Beijing, China) at 37 °C for 4 h. The relative cell viability was spectrophotometrically determined at 450 nm (Bio-Rad, USA).

### 2.3 Edu assay

BV-2 cells were incubated with Edu agent (ab219801, Abcam, Cambridge, UK) for 2 h, followed by its removal. The cells were photographed by fluorescence microscope (Axio Observer, Zeiss, Oberkochen, BW, Germany).

### 2.4 Enzyme-linked immunosorbent assay (ELISA)

After the mentioned stimulations, cell supernatants were subjected to ELISA for determining TNF-α (ab208348, Abcam, Cambridge, UK), IL-1β (ab197742, Abcam, Cambridge, UK), and IL-6 (ab222503, Abcam, Cambridge, UK) levels by following manufacturer’s guidelines. Biotin-conjugated primary antibodies were added and followed by avidin conjugated Horseradish Peroxidase (HRP). Subsequently enzyme substrate was used for the color reaction.

### 2.5 ROS assay

The cellular ROS levels were determined using 2′-7′-dichlorofluorescein diacetate (DCFH-DA, Sigma-Aldrich). Cells were washed before being analyzed by Becton Dickinson Fluorescence-Activated Cell Sorting (BD FACS) caliber as per the provided instructions.

### 2.6 Antioxidant activity detection

Superoxide Dismutase (SOD) and catalase levels were measured by the detection kits (SOD-58350 for SOD, CAT-50250 for catalase, Bio-engineering Institute, Nanjing, Jiangsu, China) according to manufacturer’s guidelines. Cells were homogenized and centrifuged (1000g) for 20 minutes and the supernatant was collected. Then the samples were added. The sample was gently shaken, mixed, and covered for reaction at 37 °C for 2 hours.

### 2.7 Immunoblot

Protein samples were separated on 10% Sodium Dodecyl Sulfate-Polyacrylamide Gel Electrophoresis (SDS-PAGE), and transferred onto Polyvinylidene Fluoride (PVDF) membranes. Proteins were blocked with 5% milk for 1 h. Primary antibodies including TNF-α (1:1000, ab183218, Abcam), IL-6 (1:1000, ab233706), IL-1β (1:500, ab216995), p-Extracellular Signal-Regulated Kinase (ERK)1/2 (1:1000, ab201015), ERK1/2 (1:1000, ab184699), p-JNK (1:500, ab215208), JNK (1:500, ab110724), p-p38 (1:1000, ab17886), p38 (1:1000, ab170099), and Glyceraldehyde 3-Phosphate Dehydrogenase (GAPDH) (1:3000, ab8245), and the secondary antibodies were incubated for 1 h. Signals were then detected using the Enhanced Chemiluminescence (ECL) kit (P0018S, Beyotime, Beijing, China).

### 2.8 Statistics

The data were analyzed by GraphPad Prism software (Version 8.0, GraphPad Software, San Diego, CA, USA). Statistical significance was determined through one-way Analysis of Variance (ANOVA), followed by Tukey’s *post-hoc* test for multiple comparisons, or Student’s *t*-test for comparing two groups. Each experiment was conducted in triplicate. Data were presented as mean ± Standard Deviation (SD). *p *< 0.05 was considered as statistically significant.

## 3. Results

### 3.1 Trazodone blocking the BV-2 cells growth

A cellular model using LPS-treated BV-2 glial cells was constructed to investigate the Trazodone effects on TN progression *in vitro*. Trazodone molecular formula is given in Fig. 1A. BV-2 cells were treated with trazodone concentrations of 0, 0.5, 1 and 2 μM for 24 h. CCK-8 assays revealed that trazodone treatment decreased the Optical Density (OD) 450 value of BV-2 cells to suggest cell growth suppression (Fig. 1B). Edu assays detected the trazodone effects on cell growth. Trazodone treatment suppressed BV-2 cells growth as shown by the decreased percentage of Edu-positive cells (Fig. 1C,D). In addition, LPS treatment suppressed the growth of BV-2 cells (Fig. 1E). Trazodone thus blocked the BV-2 cells growth.

**Fig. 1.** Trazodone blocking the BV-2 cells growth. (A) Trazodone molecular formula. (B) CCK-8 assays exhibited BV-2 cells growth upon trazodone treatment at the concentrations of 0, 0.5, 1 and 2 μM for 24 h. OD450 value was measured. (C) Edu assays exhibited BV-2 cells growth upon trazodone treatment at the concentrations of 0, 0.5, 1 and 2 μM for 24 h. Scale bar, 50 μm. (D) Quantification of panel C. Edu-positive BV-2 cells percentage was quantified. (E) CCK-8 assays exhibited BV-2 cells growth upon LPS treatment for 24 h. *p < 0.05, **p < 0.01, ***p < 0.001. TDZ: Trazodone; OD: Optical Density; LPS: Lipopolysaccharide; ns: Not Significant.

### 3.2 Trazodone suppressing the inflammation in LPS-treated BV-2 cells

Trazodone impact on the inflammation of LPS-stimulated BV-2 cells was determined. It was noticed through ELISA that LPS treatment stimulated the secretion of inflammatory factors to cause inflammation, whereas trazodone suppressed the TNF-α, IL-6 and IL-1β secretion levels in LPS-induced BV-2 cells (Fig. 2A). Expressions of these factors were detected by immunoblot. LPS upregulated the expression levels of these factors, while trazodone decreased them to suggest the inflammation suppression (Fig. 2B). Trazodone thus suppressed the inflammation in LPS-treated BV-2 cells.

**Fig. 2.** Trazodone suppressing the inflammation in LPS-treated BV-2 cells. (A) ELISA assays exhibited the secretions of TNF-α, IL-6 and IL-1β from BV-2 cells upon the treatments of LPS or trazodone at the concentrations of 0, 0.5, 1 and 2 μM for 24 h. (B) Immunoblot assays exhibited TNF-α, IL-6 and IL-1β expressions of BV-2 cells upon the treatments of LPS or trazodone at concentrations of 0, 0.5, 1 and 2 μM for 24 h. The relative expression of indicated proteins was quantified. **p < 0.01, ***p < 0.001. TDZ: Trazodone; ns: no significance; TNF-α: Tumor Necrosis Factor-alpha; IL-6: Interleukin-6; IL-1β: Interleukin-1 beta; GAPDH: Glyceraldehyde 3-Phosphate Dehydrogenase; LPS: Lipopolysaccharide.

### 3.3 Trazodone restraining the oxidative stress in LPS-treated BV-2 cells

The effects of trazodone on TN cellular model were further detected. It was found through Flow Cytometry (FCM) assays that LPS treatment, simulating the TN in BV-2 cells, upregulated the ROS levels of BV-2 cells to suggest the promotion of oxidative stress (Fig. 3A). However, trazodone decreased the ROS levels of BV-2 cells in LPS-induced BV-2 cells to suggest oxidative stress inhibition (Fig. 3A). SOD and catalase production was also detected. LPS decreased the SOD and catalase levels in BV-2 cells, while trazodone upregulated them in LPS-induced BV-2 cells to suggest the oxidative stress suppression (Fig. 3B). Trazodone thus restrained oxidative stress of LPS-stimulated BV-2 cells.

**Fig. 3.** Trazodone restraining the oxidative stress in LPS-treated BV-2 cells. (A) FCM assays exhibited ROS levels of BV-2 cells upon the treatments of LPS or Trazodone at the concentrations of 0, 0.5, 1 and 2 μM for 24 h. (B) The corresponding kits exhibited SOD (up) and catalase (down) levels of BV-2 cells upon the treatments of LPS or Trazodone at the concentrations of 0, 0.5, 1 and 2 μM for 24 h. **p < 0.01, ***p < 0.001. TDZ: Trazodone; LPS: Lipopolysaccharide; SOD: Superoxide Dismutase; FITC: Fluorescein Isothiocyanate.

### 3.4 Trazodone suppressed the MAPK pathway in LPS-treated BV-2 cells

Possible mechanism of trazodone suppressing the TN progression *in vitro* was investigated. Trazodone impact on MAPK pathway was detected by immunoblot, which mediated the cell oxidative stress and inflammation. LPS upregulated the phosphorylation levels of p38, ERK and JNK (Fig. 4). However, trazodone treatment suppressed the phosphorylation of these factors in LPS-induced BV-2 cells to suggest MAPK pathway inhibition (Fig. 4). Trazodone thus inhibited the MAPK pathway in LPS-stimulated BV-2 cells.

**Fig. 4.** Trazodone suppressed the MAPK pathway in LPS-treated BV-2 cells. Immunoblot assays exhibited the expression and phosphorylation levels of p38, ERK and JNK of BV-2 cells upon treatments with LPS or trazodone at the concentrations of 0, 0.5, 1 and 2 μM for 24 h. The relative phosphorylation levels of indicated proteins were quantified. *p < 0.05, ***p < 0.001. TDZ: Trazodone. ns: no significance; LPS: Lipopolysaccharide; ERK: Extracellular Signal-Regulated Kinase; JNK: c-Jun N-terminal Kinase; GAPDH: Glyceraldehyde 3-Phosphate Dehydrogenase.

## 4. Discussion

TN is a debilitating neuropathic pain disorder characterized by the intense periodic facial pain having impact on life quality [12]. Current treatments include pharmacotherapy with anticonvulsants such as carbamazepine and oxcarbazepine, and surgical interventions for refractory cases [13]. The efficacy of existing medications is limited because of the side effects and developing drug resistance. Novel therapeutic agents are thus imperative. Trazodone being a novel compound has emerged as potential candidate for TN treatment. This requires further research and development in addressing this painful condition.

Trazodone activity and function are being actively investigated [14]. It is primarily applied in treating psychiatric illnesses. However, recent studies have explored its potential regarding nervous system and nerve-related diseases. Trazodone has modulatory impact on CNS for managing the pain [15]. Trazodone has role in regulating the neurotransmitters, especially serotonin and dopamine [16]. It can thus alleviate pain in TN patients by reducing neural inflammation or adjusting nerve conduction. The data herein confirms that trazodone suppresses BV-2 cells growth. It restrains BV-2 cells inflammation and blocks oxidative stress.

The inhibition of glial cell proliferation by trazodone is consistent with previous studies demonstrating its neuroprotective characteristics. Inflammation and oxidative stress have roles in TN pathogenesis [15]. The inflammatory response activates pain-sensitive neurons by releasing pro-inflammatory cytokines such as tumor necrosis factor-alpha and interleukins, which lead to intensified pain sensations. Therapeutic strategies targeting oxidative stress and inflammation pathways, such as using antioxidants and regulating inflammatory responses may provide new perspectives and methods for treating TN [16]. These treatments alleviate pain and improve life quality through more conducive management options. Data herein confirm trazodone role pertaining to inflammation and oxidative stress of BV-2 cells.

This study also presents novel insights. MAPK pathway activation by trazodone in LPS-treated BV-2 cells suggest a specific mechanism where trazodone may impact the TN. Studies on the relationship between TN and MAPK pathway has provided insights into disease’s pathogenesis and potential therapeutics. MAPK pathway’s involvement in TN demonstrates its role in pain modulation and neuroinflammation [17]. Studies have demonstrated that MAPK pathway regulates processes such as histone H3 acetylation in trigeminal ganglion, effects expressions of sodium channels Nav1.8 and Nav1.9, and modulates the microglial activation in spinal trigeminal nucleus [18]. These mechanisms contribute toward the development and persistence of TN, where treatments targeting the MAPK pathway reduce pain and neuroinflammation. The identifications of molecular targets in MAPK pathway, such as Interleukin 1 Receptor Type 1 (IL1R1)/p-p38 MAPK and c-Abl-p38 pathways can provide insights of the potential therapeutic interventions to alleviate pain in TN patients. They highlight the pathway’s significance in understanding TN’s molecular mechanisms and guide about the future treatment strategies [19]. It is also revealed herein that trazodone inhibits BV-2 cell inflammation and oxidative stress by activating MAPK pathway.

This study has certain limitations which include the *in vitro* nature and focus on MAPK pathway, which may not reflect the trazodone’s impact in complex *in vivo* environments. In addition, trigeminal ganglia is indeed a better model for studying TN. The glial cells here we have chosen are relatively broad, whereas they cannot more intuitively reflect the characteristics of the *in vitro* model. Future studies must involve the animal models and clinical trials to validate these findings and explore other potential trazodone mechanisms of glial cell function and TN.

## 5. Conclusions

Trazodone inhibits glial cell hyperproliferation, inflammation, and oxidative stress through MAPK pathway activation.
